# Method for Calculating the Bending Angle of Puncture Needle in Preoperative Planning for Transjugular Intrahepatic Portal Systemic Shunt (TIPS)

**DOI:** 10.1155/2018/4534579

**Published:** 2018-05-30

**Authors:** Xiaoli Zhu, Zhao Ran, Wanci Li, Wansheng Wang, Kangshun Zhu, Wensou Huang, Xin Gao

**Affiliations:** ^1^Invasive Technology Department, The First Affiliated Hospital of Soochow University, No. 899, Pinghai Road, Suzhou, Jiangsu 215006, China; ^2^Department of Medical Imaging, Suzhou Institute of Biomedical Engineering and Technology, Chinese Academy of Sciences, No. 88, Keling Road, Suzhou, Jiangsu 215163, China; ^3^Department of Minimally Invasive Interventional Radiology, The Second Affiliated Hospital of Guangzhou Medical University, No. 250, Changgang East Road, Guangzhou, Guangdong 510260, China

## Abstract

Transjugular Intrahepatic Portal Systemic Shunt is a comprehensive interventional therapy for portal hypertension. During this intervention, puncturing from hepatic vein into portal vein is a difficult step. Selecting puncture needle with a proper bending angle is vital to accurate puncture. Thus, this prospective study provides a method to calculate the angle of the puncture needle using preinterventional contrast-enhanced CT imaging. According to the geometrical characteristics of puncture needle, Bezier curve equation was adopted to describe its bending part. By testing whether each point in a specific region satisfied the equation set of Bezier curves, the possible position of needle tip was obtained. Then, the bending angle of puncture needle was obtained by calculating curvature. The method was evaluated in 13 patients from 2 centers showing now a success rate of 100% and a duration of the procedure of 141 and 161 minutes. The method based on Bezier curve equation for calculating a proper bending angle of puncture needle was proven to be effective. And the clinical study is preliminary and additional work for clinical evaluation is necessary.

## 1. Introduction

Portal hypertension (PH) refers to a group of clinical syndromes characterized by abnormal changes of hemodynamics in portal vein system. It is also a major complication of cirrhosis and can even lead to death. Transjugular Intrahepatic Portal Systemic Shunt (TIPS) is a kind of comprehensive interventional therapy for PH [1, 2]. TIPS adopts radiation technology and uses special equipment (puncture needle with an bending angle, stent, etc.) to build a shunt between inferior vena cava and the branch of portal vein, so part of the bloodstream in portal vein can flow into inferior vena cava. Finally, portal vein pressure is decreased and severe complications caused by PH can be controlled and even prevented [3, 4].

Currently, the frequently used puncture tools are RUPS100 or RTPS100 puncture kits manufactured by Cook Company (USA). In the puncture kits, the puncture needle has only one type of bending angle. However, the causes of PH vary with patients, and the structure of intrahepatic vascular system, especially the spatial relationship between hepatic vein and portal vein, also varies with patients. Thus, the puncture needle currently used cannot meet the requirement of personalized treatment. Clinical practice indicates that choosing a puncture needle with a proper bending angle is necessary for accurate puncture of the portal vein [5]. Currently, the bending angle of puncture needle is adjusted by doctors according to their experiences. This process lacks accuracy. Once an improper bending angle is chosen, puncture cannot be performed according to the preplanned path and there is even a high risk of abdominal bleeding, resulting in intervention failure.

Therefore, the aim of this preliminary study was to provide a method that enables calculating the bending angle of the puncture needle on the basis of a previous CT imaging. The method was then evaluated in a small pilot study including 13 patients receiving a TIPS. The clinical study is preliminary and additional work for clinical evaluation is necessary.

## 2. Methods

### 2.1. Subjects

Between July 2016 and December 2016, clinical image data of 4 subjects from the First Affiliated Hospital of Soochow University (SU hospital) and 9 subjects from the Second Affiliated Hospital of Guangzhou Medical University were collected. These 13 subjects were supposed to undergo TIPS. This study was approved by the local Institutional Review Board. Among the subjects, eight patients were male and five were female. Their average age was 45 ± 4 years. Inclusion criteria include the following: (1) endoscopy confirmed esophageal or gastric varices bleeding, and the medical treatment cannot control bleeding effectively; (2) a history of portal hypertension in upper gastrointestinal bleeding and endoscopic or CT examination showed that esophageal or gastric fundus is still significantly varicose and not suitable for endoscopic therapy. Exclusion criteria included the following: (1) suffering from hepatic vein or intrahepatic portal vein or inferior vena cava occlusion; (2) patients with absolute contraindications to TIPS; (3) unwilling to accept the TIPS intervention patients

### 2.2. Path Planning

**Figure 1:**
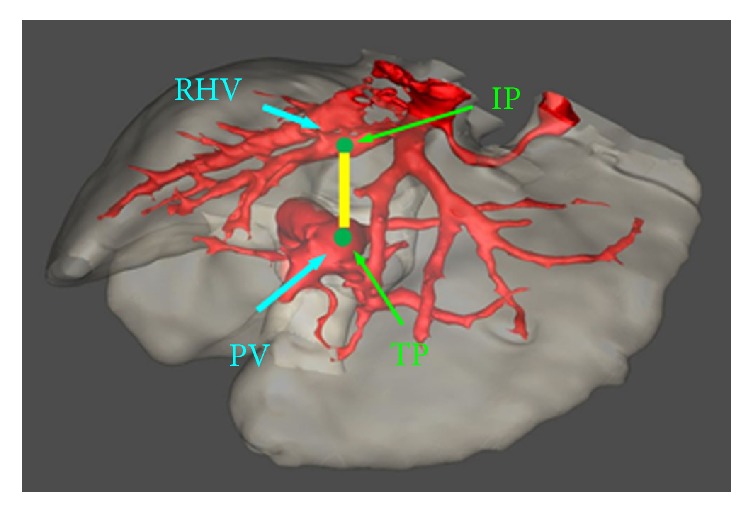
Schematic illustration of puncture path planning.

Iterative threshold segmentation combined with morphological operation was adopted to segment liver. Then, double threshold method was used to extract hepatic veins and portal veins in the segmented liver. Subsequently, 3D visualization was performed by using a surface-rendering method. Finally, the visualized image was put into 3D Slicer and an interventional physician was asked to designate two puncture points according to the anatomical spatial relationship between hepatic vein and portal vein. The point at which the needle punctured outside the hepatic vein was denoted as the Initial Point (IP). The point at which the needle punctured into the portal vein was denoted as the Terminal Point (TP). The preferred IP location was on the right hepatic veins, about 2 cm from the IVC. The preferred TP position was on the right branch of portal or on the left branch of portal, about 2 cm from the portal vein bifurcation. According to IP and TP, the puncture path was constructed (Figure 1).

### 2.3. Calculation of Bending Angle

**Figure 2:**
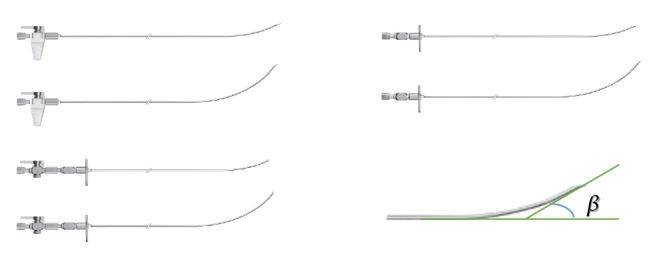
Puncture needle and its bending angle.

In the plane where the puncture path was, puncture needle moved forward from IP to TP. Puncturing into portal vein (branch) was a critical step, after which guide wire was induced into the portal vein (trunk) for subsequent stent implantation. The whole puncture process depended on the bending angle of puncture needle. Only a proper bending angle can ensure the needle to move toward portal vein after puncturing outside hepatic vein and then puncture into the branch of portal vein. Otherwise, puncture process might be performed several times before success, and the risk of liver bleeding might be increased, resulting in intervention failure. As shown in Figure 2, the angle between the tangent of needle body and the tangent of needle tip was defined as the bending angle.

#### 2.3.1. Construction of Puncture Plane

CT imaging system had self-defined coordinate system, which was denoted as S_CT_. According to parameters (voxel position, voxel size, and layer distance) in DICOM file of CT image, the coordinates of voxel in S_CT_ can be determined. The coordinates of preplanned puncture points IP and TP in S_CT_ can also be obtained, which were (*x*_*I*_, *y*_*I*_, *z*_*I*_) and (*x*_*T*_, *y*_*T*_, *z*_*T*_), respectively. A successful puncture operation meant that the puncture needle passed through IP and TP as well as a random point (RP) (*x*_*R*_, *y*_*R*_, *z*_*R*_) on the puncture path. These three points determined a puncture plane. According to equation of a plane passing through three points, that is,

(1) $$\begin{array}{c} \left|\begin{array}{ccc} x-x_{R} & y-y_{R} & z-z_{R}\\ x_{I}-x_{R} & y_{I}-y_{R} & z_{I}-z_{R}\\ x_{T}-x_{R} & y_{T}-y_{T} & z_{T}-z_{R} \end{array}\right|=0, \end{array}$$

we could obtain the equation of the puncture plane:

(2) $$\begin{array}{c} Ax+By+Cz+D=0 \end{array}$$

where A, B, C, and D were coefficients. Then, the unit normal vector of the puncture plane was $\vec{n}=(A/\sqrt{A^{2}+B^{2}+C^{2}},B/\sqrt{A^{2}+B^{2}+C^{2}},C/\sqrt{A^{2}+B^{2}+C^{2}})$, which was denoted as $\vec{n}=(n_{x},n_{y},n_{z})$.

**Figure 3:**
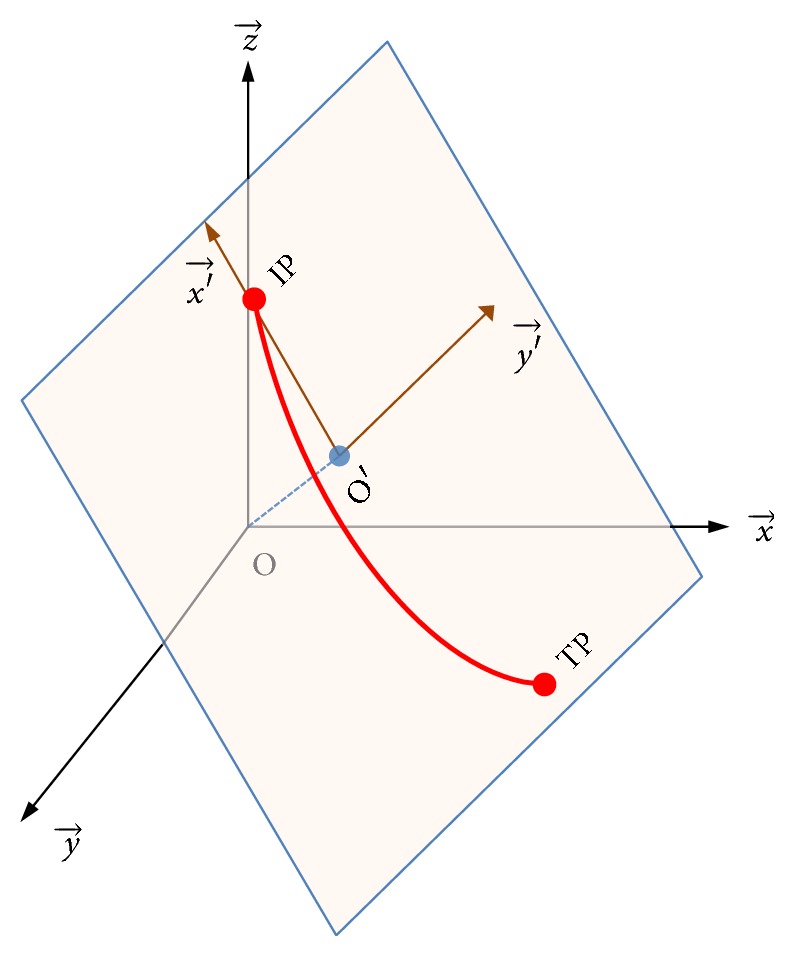
Projecting the 3D coordinate system into a 2D coordinate system. The gray plane was the puncture plane.

To conveniently use equations of curves for calculation, the 3D coordinate system was projected into a 2D coordinate system. As shown in Figure 3, the origin O of S_CT_ was projected onto the puncture plane and O′ was the projection of O. Then, O′ was taken as the origin of the new 2D coordinate system, called S_PUNC_. *x*-axis of the 2D coordinate system was the line passing through O′ and IP. *y*-axis was perpendicular to *x*-axis and also in the puncture plane. Thus, the 2D coordinate system S_PUNC_ was built for the puncture plane:

(a) Origin O′: *O*′ = −*D*(*n*_*x*_, *n*_*y*_, *n*_*z*_). Because $\vec{OO^{\prime }}//\vec{n}$, $O^{\prime }=\partial \vec{n}=\partial (n_{x},n_{y},n_{z})$. Since O′ was on the puncture plane, it should satisfy the equation of the plane (see (2)). Thus, we had $\partial =-{D/\left|\vec{n}\right|}^{2}=-D\;\;$(length of unit vector was 1).

(b) Unit vector of *x*-axis:$\;\;\vec{N_{x^{\prime }}}=\vec{O^{\prime }I}/\left|\vec{O^{\prime }I}\right|$. $\vec{O^{\prime }I}$ was the vector passing through O′ and IP. Coordinates of O′ were obtained in (a) and coordinates of IP were obtained during above-mentioned preplanning process.

(c) Unit vector of *y*-axis: $\vec{N_{y^{\prime }}}=\vec{O^{\prime }I}\times \vec{O^{\prime }O}$. Unit vector of *y*-axis was the cross product of $\vec{O^{\prime }I}$ and $\vec{O^{\prime }O}$, which was perpendicular to the puncture plane.

According to (b) and (c), the basis of S_PUNC_ was ($\vec{O^{\prime }I}/\left|\vec{O^{\prime }I}\right|$, *N*_*y*′_). Any point *Q*(*x*_*q*_, *y*_*q*_, *z*_*q*_) inside a patient's body in the original 3D coordinate system projected onto the puncture plane had coordinates $Q^{\prime }(\vec{O^{\prime }Q}\cdot \vec{O^{\prime }I}/\left|\vec{O^{\prime }I}\right|,\vec{N_{y^{\prime }}})$ in the 2D coordinate system S_PUNC_. This projection process was denoted as *f*: S_CT_→S_PUNC_.

#### 2.3.2. Curve Fitting

**Figure 4:**
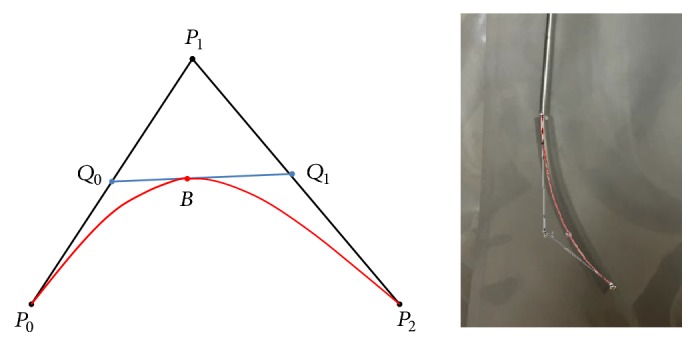
(a) Two-order Bezier curve. (b) Bezier curve matching puncture needle.

Bezier curve was an important parametric curve in computer graphics. It can use mathematical language to precisely describe any complicated graphs. Bezier curve was developed by Paul de Casteljau in 1959 using de Casteljau algorithm and was published by Pierre Bezier, a French engineer, in 1962 [6]. It had wide application in industrial design. Bezier curve had multiorder expressions. The second-order expression was defined as follows. As shown in Figure 4(a), three points including endpoints P_0_ and P_2_ and control points P_1_ were given. Point Q_0_ moved along line segment P_0_P_1_ and divided this line segment into two parts with a ratio t:1-t (first-order Bezier curve). Another point Q_1_ on line segment P_1_P_2_ was chosen and it divided the line segment P_1_P_2_ into two parts also with the ratio t:1-t. Finally, a point B on line segment Q_0_Q_1_ was chosen and it also divided the line segment P_1_P_2_ into two parts with the ratio t:1-t. When t changed from 0 to 1, Q_0_ moved from P_0_ to P_1_ and Q_1_ moved from P_1_ to P_2_. During this process, the movement trajectory of B was a second-order Bezier curve and had the following equation:

(3) $$\begin{array}{c} B\left(\;t\;\right)={\left(\;1-t\;\right)}^{2}p_{0}+2t\left(\;1-t\;\right)p_{1}+t^{2}p_{2},\;t\in \left[0,1\right] \end{array}$$

where **p**_0_, **p**_1_, and **p**_2_ are the coordinates of P_0_, P_1_, and P_2_.

It was found that Bezier curve tool in vector mapping software can well fit the bending part of the needle (Figure 4(b)). It was thus assumed that the bending part of needle can be described by the Bezier curve. In Figure 4(b), the point at which the needle began to bend (bending point) was the starting point P_0_ of Bezier curve, and the needle tip was the endpoint P_2_ of Bezier curve.

**Figure 5:**
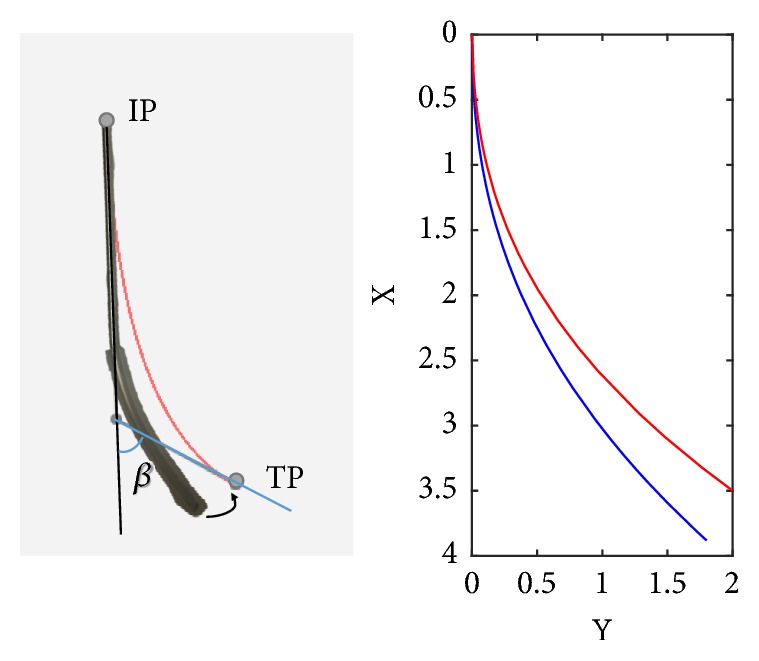
(a) Change of the bending part of puncture needle. (b) Change of corresponding Bezier curve in Mathematica.

Before intervention, the bending angle of puncture needle should be adjusted manually to meet the requirement of the preplanned path. When the bending point of puncture needle reached IP, the needle could hardly move forward. It was expected that the needle tip can reach TP. However, the currently used puncture needle had only one type of bending angle and length of the bending part was fixed. Moreover, the spatial relationship between IP and TP varied with patients. Therefore, the needle tip might not be able to reach TP. In fact, if the extension line of needle tip passed through TP, accurate puncture can also be achieved. As shown in Figure 5(a), it was assumed that the bending part of puncture needle can always be described by the equation of second-order Bezier curve when the bending angle was manually adjusted. In addition, the position of bending point and tangent at the bending point were assumed not to change, whereas the position of needle tip and tangent at the needle tip changed with the bending angle. The needle was rigid, so the length of its bending part was not supposed to change when the bending angle was adjusted. In the Bezier curve, this assumption conformed to the calculation rules. As shown in Figure 5(b), the change of the Bezier curve for puncture needle was simulated in Mathematica.

According to above assumptions, two conditions were always satisfied when the bending angle was adjusted.

(*1) The Extension Line of Needle Tip Must Pass through Puncture Point TP*. When the bending angle was proper, puncture can be successfully performed according to the preplanned path. In that case, bending point must be where IP was, and needle tip or its extension line must be able to reach TP. Bending point or IP was the starting point P_0_ of Bezier curve, and needle tip was the endpoint P_2_ of Bezier curve. The position of the needle tip changed with bending angle, but its extension line always passed through puncture point TP. Thus, the movement pattern of the needle tip can be obtained. On this basis, we could obtain the slope of tangent at needle tip according to Bezier curve and further obtain the bending angle.

The slope at any point on the Bezier curve was

(4) k=dy/dtdx/dt=−1−ty0+1−2ty1+ty2−1−tx0+1−2tx1+tx2,t∈0,1

where (*x*_0_, *y*_0_), (*x*_1_, *y*_1_), and (*x*_2_, *y*_2_) were coordinates of P_0_, P_1_, and P_2_, respectively, of Bezier curve in 2D coordinate system. When the bending angle was adjusted, (*x*_0_, *y*_0_) (i.e., coordinates of IP in S_PUNC_) remained the same, but (*x*_1_, *y*_1_) and (*x*_2_, *y*_2_) changed with bending angle. The slope at needle tip (t = 1, P2) was *k*|_*t*=1_ = (−*y*_1_ + *y*_2_)/(−*x*_1_ + *x*_2_), where (*x*_1_, *y*_1_) and (*x*_2_, *y*_2_) were unknown. The slope of line segment TP P_2_ was *k*|_*TP*,*P*_2__ = (*y*_2_ − *y*_*T*_′)/(*x*_2_ − *x*_*T*_′), where (*x*_*T*_′, *y*_*T*_′) were coordinates of TP in S_PUNC_.

(*2) The Length of Bending Part of the Needle Remained the Same*. First, we measured the length of bending part of the needle by using a soft thread. The bending part was found to have a length of 4.35 cm.

The bending part can be described by the equation of Bezier curve, so we had

(5) x′t=−21−tx0+2t1−2tx1+2tx2,t∈0,1y′t=−21−ty0+2t1−2ty1+2ty2,t∈0,1

According to integral formula

(6) $$\begin{array}{c} L=\overset{\beta }{\underset{\alpha }{\int }}1\cdot \sqrt{x^{\prime }{\left(t\right)}^{2}+y^{\prime }{\left(t\right)}^{2}}dt,\;t\in \left[0,1\right] \end{array}$$

we can obtain the length of bending part of the needle. The calculation formula was

(7) L=∫0141−t2x02+y02+41−2t2x12+y12+4t2x22+y22+8t1−2tx1x2+y1y2−81−t1−2tx0x1+y0y1−8t1−tx0x2+y0y2dt

where (*x*_0_, *y*_0_), (*x*_1_, *y*_1_), and (*x*_2_, *y*_2_) were coordinates of P_0_, P_1_, and P_2_, respectively, in 2D coordinate system.

According to the above two conditions, the following equations were obtained:

(8) ∫0141−t2x02+y02+41−2t2x12+y12+4t2x22+y22+8t1−2tx1x2+y1y2−81−t1−2tx0x1+y0y1−8t1−tx0x2+y0y2dt=4.35−y1+y2−x1+x2=y2−yT′x2−xT′

When the bending angle was adjusted, the position of starting point P_0_ did not change, whereas the positions of control point P_1_ and endpoint P_2_ changed. There were four unknown parameters but only two equations, so we cannot obtain the exact solutions of the equations. Even obtaining the movement trajectory of P_2_ (i.e., the equation that (*x*_2_, *y*_2_) satisfied) was difficult. In actual case, the range within which the bending angle changed would not be very large, since the needle was made of rigid material. Thus, we could use all the points in the circle whose center was at the initial position of the needle tip and radius was 2 cm to solve the problem. According to the resolution of the voxel space, all the points in the circle whose center was at the initial position of the needle tip and radius was 4 were tested if they satisfied (5). The step was set at 1. By doing so, we can obtain the position of P_2_. The calculation was performed in Matlab and Mathematica.

According to the definition of the bending angle, we had

(9) $$\begin{array}{c} \beta =\arctan\;{\left.\;k\;\right|}_{P_{2}}-\arctan\;{\left.\;k\;\right|}_{P_{0}} \end{array}$$

With the obtained position of P_2_ (*x*_2_, *y*_2_), we could obtain *β*.

## 3. Results

13 patients with portal hypertension underwent TIPS placement in two centers from July 2016 to December 2016. CECT was performed on each subject before TIPS. The CECT data were then used for preoperative planning.

After the original DICOM data were obtained, liver and blood vessels were segmented and were three-dimensionally visualized. Then an interventional physician was asked to designate puncture points for each subject. The coordinates of puncture points in S_CT_ for each subject were shown in Table 1.

After calculation, the bending angle of puncture needle for each subject was obtained (Table 2).

Because of a lack of intraoperative real-time imaging, the positions of actual puncture points IP and TP were a little different from those of the preplanned puncture points. Thus, the actual path was also different from the preplanned one. To evaluate the effect of bending angle on puncture efficiency, we adopted subjective and objective evaluations. The indicators were average puncture times, whether puncture needle deviated from the targeted point even punctured outside liver during intervention, whether there were puncture-related complications after intervention, and so forth. The clinical results were in Table 3.

Here, the procedure time referred to the time it took to perform the standard operations of TIPS. There were only two subjects whose results were not very good; the procedure time and puncture times of these two subjects were not as good as other patients. One was because the contrast time for CECT was improper, leading to the fact that the hepatic veins cannot be well segmented. Position of IP selected by interventional physician might be very inaccurate. The other was because the patient had undergone hepatectomy; hepatic tissue reconstruction led to difficulty in puncture.

## 4. Discussion

The bending angle of puncture needle is only one of the parameters determining the success of TIPS. Some parameters associated with the morphology of hepatic and portal venous systems as well as the positional relationship between them may also influence the accuracy and efficiency of puncture from hepatic vein to liver parenchyma and then into portal vein. However, the morphological structure of hepatic vascular system in patients with portal hypertension of cirrhosis is abnormal. This is caused by the atrophy of liver parenchyma and liver ascites after hepatic fibrosis. Thus, the puncture needle bending angle that we calculated is a reference value. Applying our method, probably many punctures will still not be successful because the needle bending may change during advancing the needle and the liver may move and twist during the puncture. Moreover, the procedure does not provide real-time guidance. Therefore, this does not replace sonographic real-time 3D guidance. It is definitely the gold standard. However, our method can be used as an adjunct, which may always improve the technique irrespective of other guiding attempts.

TIPS is a very difficult and highly risky intervention. In fact, it is thought to be one of the most complicated interventions by* Peripheral Vascular Interventional Diagnosis and Surgery Hierarchical Directory*. The process of puncture needle puncturing outside hepatic vein in a specific region and then precisely puncturing into portal vein branch is a difficult step of TIPS. To solve this problem, Image-guided TIPS intervention [7–9] and path planning method [10–13] have been developed. However, none of them performed calculation of the bending angle of puncture needle. In fact, the bending angle of puncture needle was often subjectively adjusted because of the lack of precise calculation, which might lead to the deviation of puncture needle from the targeted point and the damage to other tissues. In that case, the puncture needle needed to be taken out and reinserted in again, resulting in more puncture times. This paper proposed a method based on Bezier curve equation to precisely calculate a proper bending angle of puncture needle for TIPS. Bezier curve was used to describe the bending part of puncture needle, and Bezier curve equation was used to solve the problem of bending angle. Adjusting puncture needle to obtain a proper bending angle was a critical step during preoperative planning.

A path planning method based on numerical calculation model was proposed, where the critical puncture points can be selected according to preoperative 3D CECT. In addition, a proper bending angle of puncture needle can be calculated based on Bezier curve equation.

Clinical results showed that our method for adjusting the bending angle of puncture needle can well guide the puncture of the portal vein. In this case, the preplanned puncture path and the precisely calculated bending angle of puncture needle can improve puncture accuracy and reduce puncture times, ensuring an effective puncture operation. Knowledge of the bending angle has its own advantage whatever additional guidance is used.

The clinical study is preliminary; the method should be further evaluated intraoperatively and postoperatively on more cases. In the future, we will evaluate the performance of this method by using it in more clinical trials from multiple centers and will propose methods for calculating other parameters related to 3D puncture path.

## Figures and Tables

**Table 1:** Coordinates of puncture points in S_CT_ chosen by interventional physician for each subject.

Subject number	IP (*x*_*I*_, *y*_*I*_, *z*_*I*_)	TP (*x*_*T*_, *y*_*T*_, *z*_*T*_)
1	(218,263,34)	(190,230,24)
2	(188,243,34)	(172,240,25)
3	(170,275,35)	(172,258,27)
4	(202,258,27)	(201,251,21)
5	(192,241,31)	(172,228,21)
6	(225,285,34)	(210,263,23)
7	(204,258,37)	(201,248,30)
8	(201,255,72)	(197,236,65)
9	(201,332,27)	(175,317,21)
10	(202,262,37)	(188,257,29)
11	(210,265,36)	(197,248,30)
12	(201,264,33)	(189,257,27)
13	(210,283,33)	(189,257,27)

**Table 2:** Calculated bending angle of puncture needle for each subject.

Subject number	Bending angle (degree)
1	30.7
2	41.0
3	37.5
4	36.2
5	39.5
6	40.2
7	66.7
8	52.5
9	54.2
10	36.0
11	36.2
12	45.1
13	41.0

**Table 3:** Clinical results of subjects in two research centers.

Research center	Average puncture times	Average procedure time (min)	Puncture outside liver	Puncture-related complications
SU^1^ (n=4)	3	161	1	0
SYSU^2^ (n=9)	2	142	0	0

^1^SU refers to SU Hospital.

^2^SYSU refers to SYSU Hospital.

## Data Availability

The data presented in this study are provided by the First Affiliated Hospital of Soochow University and the Second Affiliated Hospital of Guangzhou Medical University. For the acquisition of raw data, please contact the corresponding author Professor Xin Gao at xingaosam@yahoo.com.
